# Bone marrow niche-mimetics modulate HSPC function via integrin signaling

**DOI:** 10.1038/s41598-017-02352-5

**Published:** 2017-05-31

**Authors:** Martin Kräter, Angela Jacobi, Oliver Otto, Stefanie Tietze, Katrin Müller, David M. Poitz, Sandra Palm, Valentina M. Zinna, Ulrike Biehain, Manja Wobus, Triantafyllos Chavakis, Carsten Werner, Jochen Guck, Martin Bornhauser

**Affiliations:** 1 Medical Clinic I, University Hospital Carl Gustav Carus, Dresden, Saxony 01307 Germany; 2 Biotechnology Center, Technische Universität Dresden, Dresden, Saxony 01307 Germany; 3 Centre for Innovation Competence - Humoral Immune Reactions in Cardiovascular Diseases, University of Greifswald, Greifswald, Mecklenburg-Western Pomerania 17489 Germany; 4 Department of Internal Medicine and Cardiology, Technische Universität Dresden, Dresden, Saxony 01307 Germany; 5 Department of Clinical Pathobiochemistry, Technische Universität Dresden, Dresden, Saxony 01307 Germany; 6 Institute for Clinical Chemistry and Laboratory Medicine, Technische Universität Dresden, Dresden, Saxony 01307 Germany; 7 Center for Regenerative Therapies Dresden, Technische Universität Dresden, Dresden, Saxony 01307 Germany; 8 Leibniz Institute of Polymer Research Dresden, Max Bergmann Center of Biomaterials, Dresden, Saxony 01307 Germany

## Abstract

The bone marrow (BM) microenvironment provides critical physical cues for hematopoietic stem and progenitor cell (HSPC) maintenance and fate decision mediated by cell-matrix interactions. However, the mechanisms underlying matrix communication and signal transduction are less well understood. Contrary, stem cell culture is mainly facilitated in suspension cultures. Here, we used bone marrow-mimetic decellularized extracellular matrix (ECM) scaffolds derived from mesenchymal stromal cells (MSCs) to study HSPC-ECM interaction. Seeding freshly isolated HSPCs adherent (AT) and non-adherent (SN) cells were found. We detected enhanced expansion and active migration of AT-cells mediated by ECM incorporated stromal derived factor one. Probing cell mechanics, AT-cells displayed naïve cell deformation compared to SN-cells indicating physical recognition of ECM material properties by focal adhesion. Integrin αIIb (CD41), αV (CD51) and β3 (CD61) were found to be induced. Signaling focal contacts via ITGβ3 were identified to facilitate cell adhesion, migration and mediate ECM-physical cues to modulate HSPC function.

## Introduction

Hematopoietic stem and progenitor cells (HSPCs) are anchored in a specialized microenvironment in the bone marrow (BM) called the hematopoietic niche^1–3^. These cells are defined by their self-renewing capacity and their ability to give rise to all mature blood cells^4, 5^. Human HSPCs can be enriched via the surface antigen CD34 before clinical or tissue engineering use^6^. Since, these cells represent a minority in most graft sources and the amount of applicable cells is limited, *ex vivo* expansion-cultures have been established using cytokine cocktails^7–9^ or small molecules^10^. However, *in vitro* culture of HSPCs in suspension leads to heterogeneous cell-populations with undefined cellular identities^11^.

In the BM niche HSPCs are not exclusively maintained by cytokines but preliminary by cell-matrix adhesion mediated via adhesion receptors, such as integrins (ITGs). In this regard, β1 (CD29) and β2 ITGs were found to promote the initial contact of HSPCs to mesenchymal stromal cells (MSCs)^12^ and β3 (CD61) expression was shown to be a marker for long-term repopulating HSPCs *in vivo* ^13, 14^. Consequently, remodeling the BM niche *ex vivo* using co-cultures of HSPCs and niche cells like MSCs fade into spotlight and was proven to be a promising tool for stem cell expansion^15–18^. However, in clinical or research applications direct contact of two cell populations necessitates HSPC post-culture purification.

To face these problems, we used a novel culture method remodeling the BM extra cellular stroma *in vitro*, namely decellularized extracellular matrix (ECM) scaffolds derived from immortalized mesenchymal stromal cells (SCP-1)^19^. Such scaffolds were found to be highly reproducible and provide >500 proteins including major niche proteins like collagens, fibronectin, glycosaminoglycans as well as osteopontin and signaling molecules like stromal derived factor 1 (SDF-1). Thus, adhesive sides and physical cues as they are present in the BM niche^20^ are provided. Biomechanical forces of the surrounding environment were found to be transduced to cells via integrin signaling^21^ and were demonstrated to control HSPC fate *in vitro* ^22–24^. These physical aspects were shown to be a marker for differentiation^25^ and drive cell fate decision^26, 27^. However, the detailed mechanisms of HSPC interaction with the BM stroma regarding exclusively the extracellular environment is less understood.

By culturing human CD34^+^ cells isolated from granulocyte colony-stimulating factor (G-CSF) mobilized peripheral blood (PB) of healthy donors with low concentrations of cytokines on decullarized ECM preparations we could separate two cell fractions: adherent (AT) and supernatant (SN) cells, similar to HSPC co-cultures with MSC^28^. Using real-time deformability cytometry (RT-DC)^29^ to uncover biomechanical phenotypes of freshly isolated, SN, AT and classical suspension cultured HSPCs in plastic culture dishes (PCD), we found freshly isolated cells to be homogenous deformable and small compared to cultured cells. AT-cells revealed actin polymerization to stress fibers, strong migratory behavior and a less deformable phenotype compared to PCD cultured or SN-cells. SDF-1 was found to be actively recognized and internalized by AT-cells through C-X-C chemokine receptor type 4 (CXCR4). Additionally, CXCR4 was seen to be polarized towards ECM proteins in AT cells. Most importantly, we found induction of surface expression of ITGαIIb, ITGαV and ITGβ3 on AT-cells and could confirm the important role of ITGαvβ3 for HSPC-ECM interaction with regard to adhesion and migration.

## Results

### ECM promotes HSPC survival and expansion

**Figure 1:**
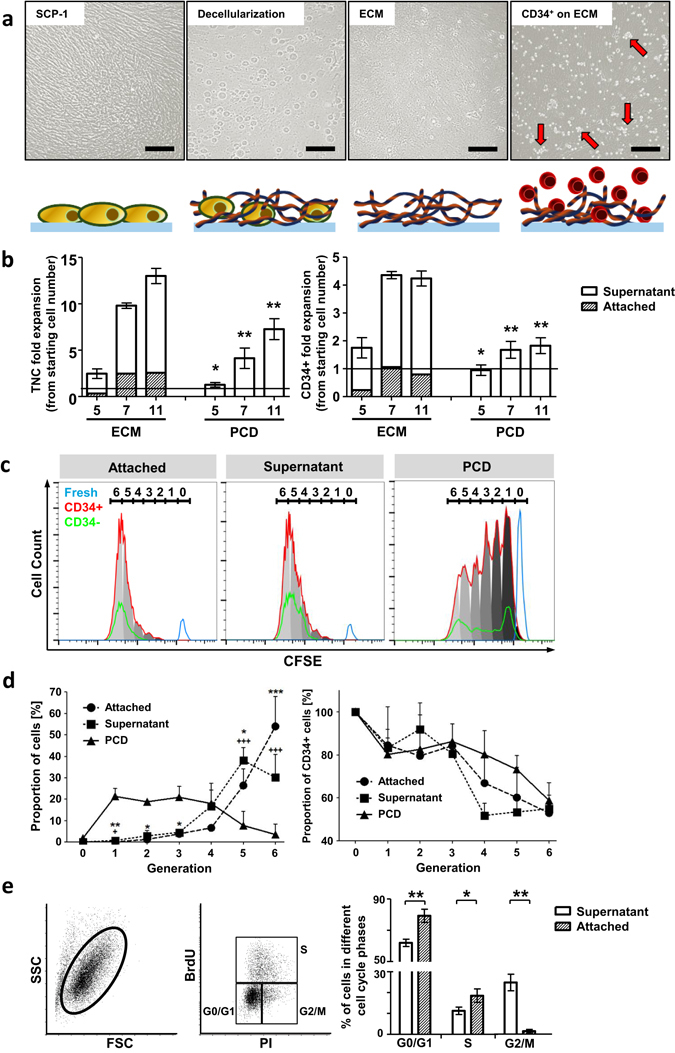
ECM scaffolds support CD34+ cell expansion *in vitro*. (**a**) Phase contrast images and corresponding cartoons of decellularization process for ECM scaffold generation. After 5 days in ECM-culture cells showed clustered accumulation (red arrows) (**b**) *In vitro* TNC and CD34+ cell expansion either on ECM or PCD culture for 5, 7 and 11 days. Fold change in relation to starting cell number. AT-cells are presented as proportion of ECM cultured cells. n = 5, two-tailed t-test, significance in comparison to ECM (**c**) Representative CFSE-intensity histogram after 5 days in ECM or PCD culture representing distribution of cell generations of CD34+ (red) and CD34- (green) cells. Fresh cells (blue) as control (generation 0). (**d**) Proportion of 5 days ECM and PCD cultured cells (left) or CD34+ (right) cells in cell generations. n = 3, two-tailed t-test; * = AT-cell and + = SN-cell significance in comparison to PCD. (**e**) Representative flow cytometry dot-plots for BrdU- and PI-staining (left panel). Quantification of cell cycle phases of SN- and AT-cells after 5 days ECM culture (right panel). n = 3, two-tailed t-test. Error bars, s.e.m.; *p < 0.05, **p < 0.01, ***p < 0.001.

To mimic the HSPC-niche *ex vivo* we used MSC (SCP-1)-derived decellularized ECM scaffolds as culture substrates. Decellularized ECM quality was assessed and protein structure was visualized using inverted microscopy (Fig. 1a). After seeding purified CD34^+^ cells from mobilized PB in serum-free CellGro medium using ultra low cytokine concentration (2.5 ng/ml each) we observed clustered adhesion of HSPCs to the underlying substrate after less than 12 h (Fig. 1a). However, just 20% of all seeded CD34^+^ cells were adherent on the presented ECM-proteins (Fig. 1a). This proportion of AT-cells was found to be constant over culture and expansion time. Both adherent (AT) and non-adherent (SN) cell populations, were found to actively proliferate under ECM culture conditions. After 5 days, total nucleated cells (TNCs) expanded up to 3 fold, which represents a significantly higher expansion compared to PCD cultures (1.5 fold, p < 0.05). By increasing culture periods for 7 or 11 days, TNC number cultured on ECM increased in average by 7.2 fold and 13 fold, respectively. Interestingly, the amount of AT-cells did not further increase after 7 days. Using flow cytometry, we found ECM scaffolds to significantly expand CD34^+^ progenitor cells (by 1.8 fold), as compared to maintenance of CD34^+^ cell numbers in PCD control cultures after 5 days (p < 0.05) (Fig. 1b). After removing SN-cells we monitored proliferation of AT-cells and repopulation of the supernatant fraction, indicating further division but no increased adhesion (data not shown). Similar findings were presented by Jing *et al*. 2010 using a MSC-HSC co-culture *in vitro* niche model^18^.

To analyze the division history of HSPCs, CFSE staining was applied to freshly isolated CD34^+^ cells and flow cytometry was used to detect HSPC generations either in 5 days ECM- or PCD-cultures. Generation 0 was represented by freshly isolated cells. We detected up to 6 generations in both culture conditions. However, ECM cultured cells had mainly undergone 5 to 6 divisions (Fig. 1c) and up to 80% of AT-cells or 70% of SN-cells were found in generation 5 and 6 (Fig. 1d). On the contrary, only approximately 10% of PCD cultured cells were found in these generations in line with the aforementioned lower expansion rate (Fig. 1d). Surprisingly, AT-cells showed the highest proliferation and up to 55% of all cells divided 6 times compared to 30% of SN-cells. According to BrdU incorporation assays, after 5 days AT-cells were found mainly in cycling G0/G1 (79.3% ± 5.4%) and S-phase (18.5% ± 3.9%). Significantly less cells could be detected in G2/M (1.9% ± 1.4%) compared to SN-cells (G0/G1 63.4 ± 3.4%, p < 0.01; S 11.8% ± 2.9%, p < 0.05; G2/M 25.4% ± 6.8%, p < 0.01) (Fig. 1e).

### AT-cells display strong actin polymerization and a native mechanical phenotype

**Figure 2:**
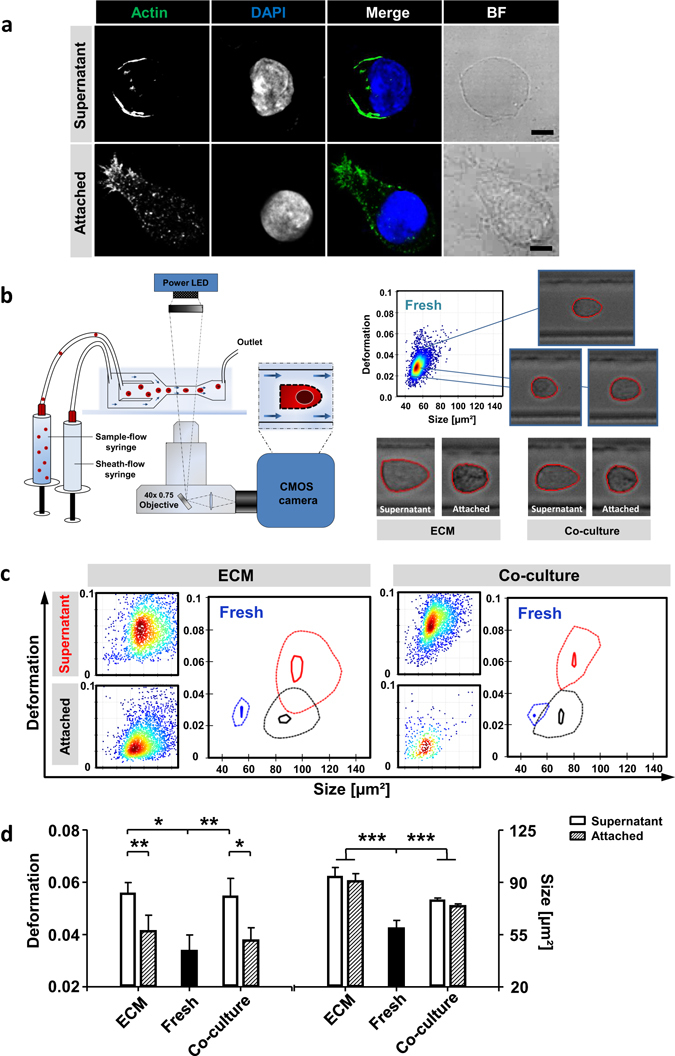
HSPC adhesion to ECM-proteins remodel morphological and mechanical phenotype. (**a**) Confocal microscopy single z-slice images of actin cytoskeleton using phalloidin-488 (green) staining and nuclei DAPI (blue) staining. Bars = 5 µm. (**b**) Schema shows an overview of RT-DC set-up. Representative heat-plot of RT-DC measurement was recorded directly after MACS isolation of fresh CD34+ cells. Representative images of cells in microfluidic channel constriction of fresh, ECM or MSC co-cultured cells. (**c**) Representative heat-plots of cell-size and cell-deformation of AT- and SN-cells either ECM- or MSC co-cultured. Contour-plots compare freshly isolated cells (blue), AT-cells (black) and SN-cells (red). The solid inner line highlights 95% and the dashed outer line 50% density. Cells measured after a 5 day cultivation period. (**d**) Statistical analyses of deformation and cell-size measured in RT-DC of fresh cells and ECM- or MSC co-cultured cells. n = 5, linear mixed model analysis, Error bars, SD.; *p < 0.05, **p < 0.01, ***p < 0.001.

Since contact to ECM-scaffold or MSC feeder-layer strongly influences HSPC shape^16, 30^ we investigated how SN- and AT-cells differ in terms of morphology and physical parameters. To analyze cytoskeletal arrangement we performed immunofluorescence staining of filamentous actin (F-actin) (Fig. 2a). Thereby, we found F-actin towards a polarized morphology in ECM-cultured (Fig. 2a) and MSC co-cultured (Fig. S1a) AT-cells.

For biophysical characterization in RT-DC, shear stress was applied to cells in a microfluidic channel constriction and deformation and cell-size were recorded in real-time. This technique is based on image processing and allows an optical validation of cell shape and morphology (Fig. 2b, left cartoon). In Fig. 2b (right) representative images of freshly isolated cells as well as ECM cultured and MSC co-cultured HSPCs are shown when passing the channel constriction. Interestingly, ECM cultured and MSC cultured AT-cells exhibited macroscopically denser intracellular packaging and accumulation of granular structures compared to SN-cells, which might be associated with the aforementioned F-actin structure (Fig. 2b). Freshly isolated cells were found to be homogeneous in cell-size (56.6 ± 7.05 µm²) and deformation (0.034 ± 0.006) thereby clearly distinguishable from *in-vitro* expanded cells. Upon culture on ECM scaffolds, cells became larger with an average cell size of approximately 104.1 ± 7.3 µm². In contrast, MSC co-cultured cells did not grow equivalently revealing an average cell-size of 80.8 ± 1.4 µm² (p < 0.001) (Fig. 2c). However, cell-size of ECM scaffold (p < 0.001) and MSC co-cultured (p < 0.001) cells significantly differed from freshly isolated CD34^+^ cells (Fig. 2c,d right chart). For active proliferation in ECM culture and MSC co-culture, cells had to grow before subsequent cell division. By comparing the deformability of ECM scaffold cultured AT- and SN-cells AT-cells showed a significantly less deformable phenotype (d = 0.041 ± 0.006) compared to SN-cells (d = 0.056 ± 0.003; p < 0.01). This was also true in MSC co-cultured cells (AT-cells, d = 0.034 ± 0.005; SN-cells, d = 0.055 ± 0.007; p < 0.05) (Fig. 2c,d left chart). Interestingly, the deformation of AT-cells did not significantly differ from freshly isolated CD34^+^ cells (d = 0.034 ± 0.006). In contrary, deformation of SN-cell remarkably increased in comparison to freshly isolated cells (p < 0.001) (Fig. 2d,c). Nevertheless, SN-cells are comparable to classical PCD suspension cultured cells with regard to cell size and deformation (Fig. S1b).

Our data indicate that adhesion to ECM-proteins remodel HSPCs towards a more naïve morphological and mechanical phenotype, pointing to functional equality.

### SDF-1 retains its bioactive conformation on decellurarized ECM

**Figure 3:**
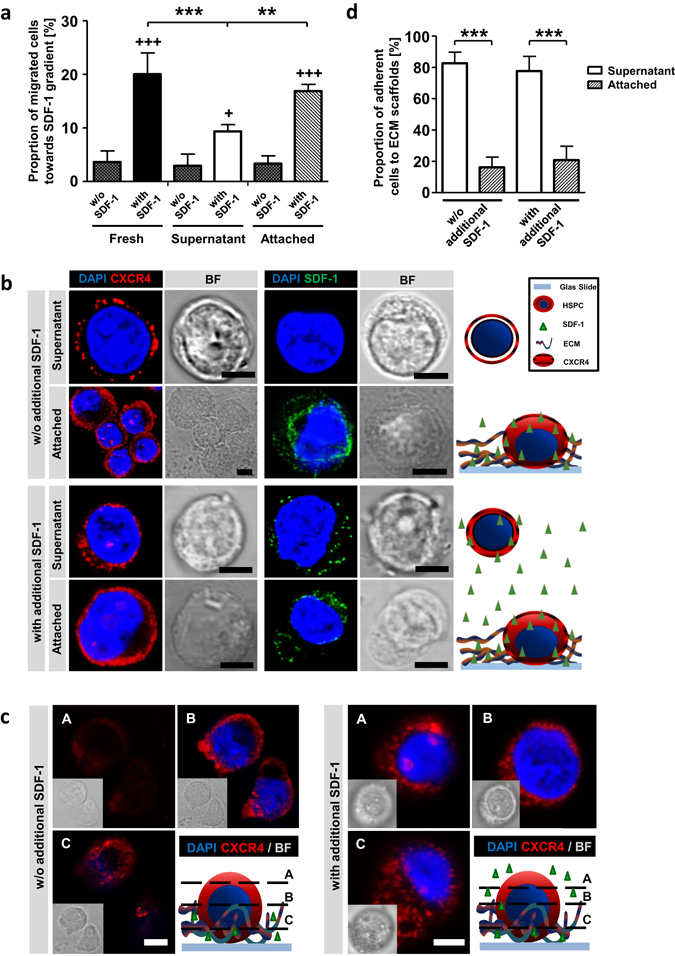
SDF-1/CXCR4 axis is active on ECM cultured HSPCs. (**a**) Proportion of migrated fresh-, SN- and AT-cells in a trans-well migration assay towards SDF-1 containing medium (with SDF-1) or towards SDF-1 free medium (spontaneous migration, w/o SDF-1). n = 4, two-tailed t-test; * = significance in comparison to SN-cells and + = significance in comparison to corresponding w/o SDF-1. (**b**) Confocal microscopy images of CXCR4 (red) or SDF-1 (green) staining and nuclear DAPI (blue) staining. Cells were treated with medium either without (w/o) or with additional SDF-1. Schemas show technique and findings. Bars = 5 µm. (**c**) Confocal microscopy z-stack images of α-CXCR4 (red) and nuclei DAPI (blue) staining. A = top, B = middle and C = bottom of cells. Cells were treated with medium either without or with additional SDF-1. Schemas show technique and findings. Bars = 5 µm. (**d**) Proportion of cell adhesion to ECMs 24 hours after seeding either without or with additional SDF-1 containing medium. n = 6 for w/o additional SDF-1, n = 3 for with additional SDF-1, two-tailed t-test. Error bars, SD.; *p < 0.05, **p < 0.01, ***p < 0.001.

The SDF1/CXCR4 axis is the most commonly investigated chemokine axis in hematology and is known to be essential for homing and retention of HSPCs in the BM^31, 32^. Therefore, we asked whether SDF-1 could also be involved in the adhesion to ECM preparations. Using a trans-well migration system we aimed at determining the ability of freshly isolated CD34^+^ cells, AT-cells and SN-cells to migrate along a SDF-1 gradient. We found that 20.1% ± 3.4% of freshly isolated cells actively migrated towards SDF-1 containing medium (spontaneous migration 3.6% ± 1.8%), which is in line with reported findings^33^. AT- and SN-cells, however, significantly differed in their potential to migrate towards SDF-1. 16.9% ± 1.1% of AT-cells were found to migrate within 3 h, whereas only 9.9% ± 1.1% of SN-cells reached the bottom chamber (p < 0.01). Spontaneous migration was measured to be less than 3.5% in AT- and SN-cells (Fig. 3a).

We have previously demonstrated that amongst various morphogens, SDF-1 can be retrieved from decellularized ECMs derived from human BM-MSCs^34^. To test whether SCP-1 cells also secrete bioactive SDF-1 anchored by ECM-proteins we performed immunostainings for SDF-1 and CXCR4 on AT- and SN-cells after 5 days of culture. Using confocal microscopy, we detected CXCR4 expression in AT- and SN-cells. Whereas SN-cells showed a predominantly membranous expression pattern, AT-cells seem to exhibit intracellular staining (Fig. 3b, upper panel). However, due to a low cytoplasm to nucleus ratio in SN-cells, a detailed subcellular localization of CXCR4 is hardly possible. Interestingly, only AT-cells showed SDF-1 internalization, representing active recognition and uptake from ECM proteins (Fig. 3b). In order to assess SDF-1 processing of SN-cells, we added exogenous chemokine in varying concentrations. In these experiments, CXCR4 and SDF-1 were found to be located in the cytosol of SN-cells, indicating internalization of ligand-receptor-complex by active recognition of recombinant SDF-1 (Fig. 3b). However, additional SDF-1 did not influence the amount of AT-cells (Fig. 3d). Using Z-stack imaging in confocal microscopy on HSPCs (A = top, B = middle, C = bottom) we detect highest amount of CXCR4 on the bottom of cells during contact with ECMs (Fig. 3c, left panel), which was the same for SCP-1 co-cultures (Fig. S2). This polarization was suppressed when additional SDF-1 was added to the culture medium (Fig. 3c, right panel). These findings reveal incorporation of bio-active SDF-1 into ECM scaffolds and recognition via CXCR4.

### Adhesion is promoted via RGD axis ITGβ3, ITGαV and ITGαIIb

**Figure 4:**
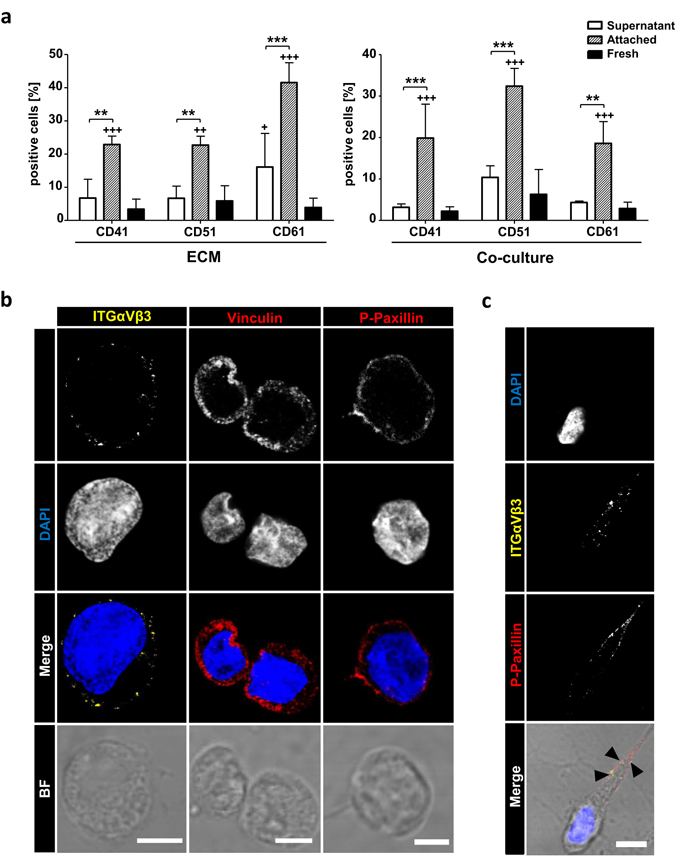
Integrins recognizing RGD-motives promotes active focal contact formation. (**a**) FACS analysis of freshly isolated cells or 5 days ECM or MSC co-cultured cells. Percent positive cells for ITGαIIb (CD41), ITGαV and ITGβ3 (CD61) are shown. n = 4, two-way ANOVA with Bonferroni post-hoc test; + = significance in comparison to fresh cells. Error bars, s.e.m.; *p < 0.05, **p < 0.01, ***p < 0.001. (**b**) Confocal microscopy images of α-ITGαVβ3 (left panel, yellow), α-vinculin (mid penal, red), α-p-paxillin (right panel, red), nuclei DAPI (blue) staining, merge and corresponding brightfield images of 5 days cultured AT-cells. Bars = 5 µm. (**c**) Confocal microscopy images of α-ITGαVβ3 (yellow), α-p-paxillin (right panel, red), nuclei DAPI (blue) staining and merge including corresponding brightfield image of 5 days cultured AT-cell. Arrowheads represent co-localization of ITGαVβ3 and p-paxillin in a cellular protrusion. Bar = 5 µm.

Since integrins mediate cell matrix contact in BM^35^, we asked whether integrin expression differed on fresh -, AT- and SN-cells. We used flow cytometry to identify surface expression of HSPC related integrins (CD29, CD41, CD49d, CD49e, CD49f, CD51 and CD61) (Table 1). Remarkably, 22.9% ± 6.3% of AT-cells expressed ITGαIIb, whereas only 6.7% ± 5.2% of SN- and 3.4% ± 2.8% of freshly isolated cells were ITGαIIb-positive. Moreover, 22.7% ± 6.6% of AT-cells showed ITGαV expression and 41.5% ± 14.7% were found to be positive for ITGβ3. In contrast, 6.7% ± 3.4% and 16.1% ± 9.4% of SN-cells were positive for ITGαV and ITGβ3, respectively. Accordingly, a minority of freshly isolated cells 5.8% ± 4.2% and 3.9% ± 2.6% were found positive for ITGαV and ITGβ3, respectively (Fig. 4a, ECM). Slightly different results were found for MSC co-cultured HSPCs. Here, increased numbers of ITGαV^+^ cells were encountered, however, the effect was not as pronounced as on ECMs (Fig. 4a, Co-culture).

**Table 1:** Integrins described as HSPC related.

Integrin	CD	Gene Name	Ligands (in heterodimeres)	HSPC Function
α4	CD49d	ITGA4	Fibronectin VCAM-1	BM homing, BM engraftment, maintenance blocking leads to mobilization^35, 63^
α5	CD49e	ITGA5	Fibronectin Osteopontin	BM lodgement via osteopontin binding^64, 65^
α6	CD49f	ITGA6	Laminin	Blocking leads to reduced BM homing^66^
αIIb	CD41	ITGAIIb	Fibronectin	Early hematopoiesis, BM logement, regulation of progenitor number^67–69^
αV	CD51	ITGAV	Fibronectin Vitronectin Tenascin	Maintenance^37^
β1	CD29	ITGB1	Collagen Laminin Fibronectin Osteopontin	BM lodgement, Differentiation^12, 70^
β3	CD61	ITGB3	Fibronectin	Maintenance^13, 14, 37^

Interestingly, the identified candidates are known to form RGD-motive recognition dimers^36^. We interrogated whether a combination of ITGαV and ITGβ3 is expressed on AT-cells, which is described as a regulator of HSPC engraftment and maintenance by forming focal contacts and promoting outside-in (ECM to cell) signaling^37^. Using confocal microscopy we identified ITGαVβ3 as membrane associated in AT-cells (Fig. 4b). Nevertheless, ITGαVβ3 could not be detected in SN-cells (data not shown). To prove active focal adhesion formation we performed further immunostainings including vinculin and phosphorylated paxillin. Vinculin was found to be highly expressed in AT-cells and located in the cytosol as well as membrane associated (Fig. 4b). AT- and SN-cells expanded rapidly during ECM culture. Hence, we also found vinculin expression in SN-cells (Fig. S3), as vinculin is indispensable for HSPC repopulation^38^. Paxillin, in contrast, was found to be phosphorylated at Tyr118 when membrane associated (Fig. 4b) and localized near ITGαVβ3 exclusively in AT-cells (Fig. 4c, arrowhead). This indicates formation of activated signaling focal contacts through integrin mediated adhesion to ECM proteins.

### Inhibition of ITGαVβ3 leads to reduced HSPC migration

**Figure 5:**
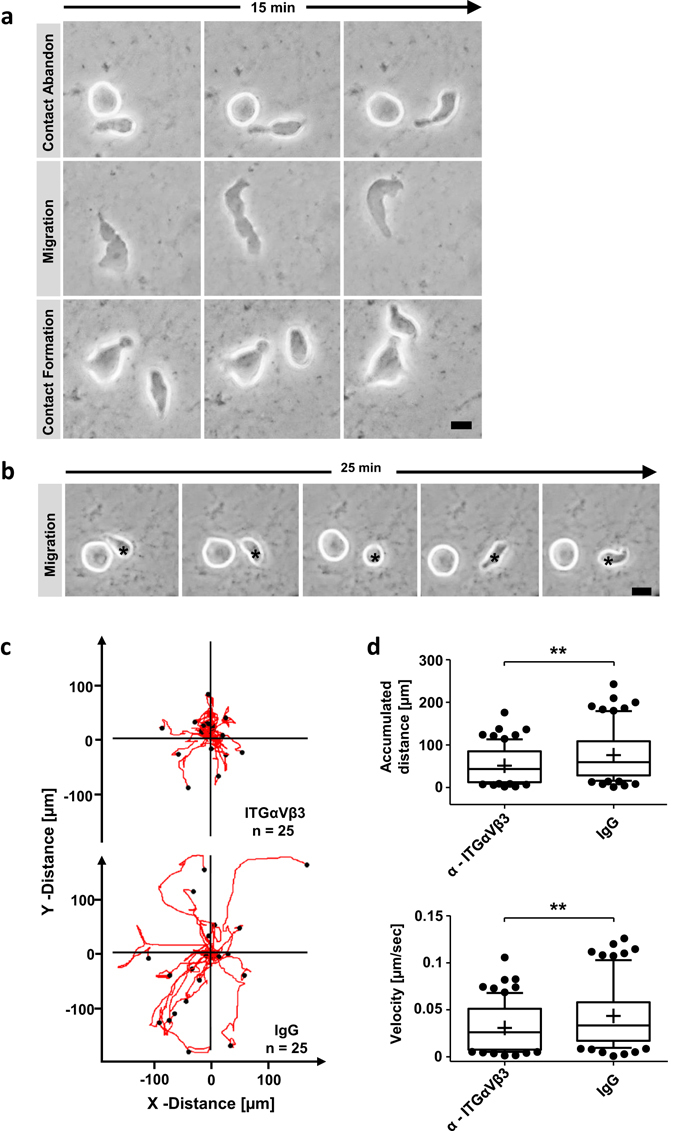
Blocking ITGαVβ3 attenuates migration of HSPCs. (**a**) Individual frames of 15 minutes time-laps microscopy studies of migrating and interacting cells on ECMs. Bar = 5 µm. (**b**) Individual frames of 25 minutes time-laps microscopy of a non-migrating cell and a migrating cell forming no-migrating phenotype (asterisk). Bar = 5 µm. (**c**) Cells were cultured 5 days on ECMs and incubated one hour with α-ITGαVβ3 or IgG. Trajectory plots depict migration of 25 HSPCs for 45 minutes. (**d**) Box-and-whiskers plots presenting accumulated distance and velocity of 77 HSPCs migration tracks monitored for 45 minutes pre-incubated 1 hour with α-ITGαVβ3 or IgG. Lines show 25th – 75th percentiles, horizontal line represents median and plus represents mean value. two-tailed t-test.; *p < 0.05, **p < 0.01, ***p < 0.001.

When cells adhere to ECM preparations they highly interact by active migration (Fig. 5a). In this context, migrating and non-migrating cells adopt various polarized and non-polarized morphologies. In our study, migrating cells displayed a flattened and elongated shape and formation of lamelliopodia towards the direction of migration (Fig. 5a, Movie S1). Non-migrating cells were found to retain a spherical shape forming microvilli-like projections around the cell (Movie S1). This is in line with HSPCs in contact with either human or murine MSCs^39^. However, ECM cultured cells are not fixed in these states. Using time-lapse microscopy we found plasticity of individual AT-cells between migratory and non-migratory phenotypes during an observation period of 25 minutes (Fig. 5b). These dynamics were characterized by migration (migratory phenotype), resting (non-migratory phenotype) and continued migration (Fig. 5b, asterisk).

Since we found ITGαVβ3 predominantly expressed and forming focal contacts on AT-cells we asked whether this integrin heterodimer promotes migration. Using either a blocking antibody for ITGαVβ3 or an appropriate IgG control we monitored adherent cells for 45 minutes. The average migration distance and velocity was calculated including all cells per section (Movie S1). In both conditions the ratio of cells displaying migratory or non-migratory phenotype was not changed (Fig. S4a,b). However, AT-cells incubated with blocking antibody migrated significantly slower with an average velocity of 0.026 μm/s ± 0.02 μm/s compared to control treated cells with an average velocity of 0.038 μm/s ± 0.03 μm/s (p < 0.01) (Fig. 5d). In this context, the accumulated distance decreased when AT-cells were incubated with ITGαVβ3 blocking antibody (60.6 μm ± 49.3 μm) compared to IgG treated cells (86.8 μm ± 65.5 μm; p < 0.01) (Fig. 5c,d). Interestingly, an effective inhibition of cell migration using the blocking antibody could still be achieved 24 h after seeding HSPCs on ECM substrates. Nevertheless, cells showed in general less migratory behavior (Fig. S4c), which points to ITGαVβ3 as an essential ECM contact mediating integrin with importance for cell migration.

### ITGβ3 expression is induced by ECM contact

**Figure 6:**
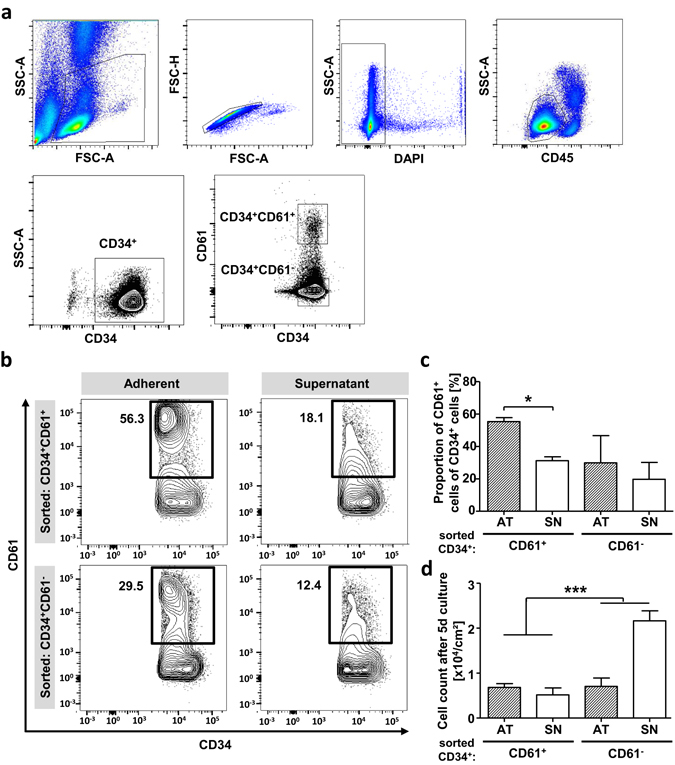
ECM scaffolds induce CD61 surface expression on HSPCs. (**a**) Representative gating strategy for fluorescence activated cell sorting experiments. Lymphocytes and single cells were enriched using light-scatter. Dead cell exclusion was done by DAPI staining. Progenitor enrichment was done using SSC-Alow/CD45dim and CD34+ gate. Sorting gates CD34+CD61- and CD34+CD61+. (**b**) Representative FACS plots of CD34+CD61+ and CD34+CD61- sorted fresh cells after 5 days in ECM culture. AT- and SN-cells are shown regarding CD34 and CD61 expression using gates according to sort experiments. (**c**) FACS analysis of CD34+CD61+ and CD34+CD61- sorted cells after 5 days in ECM culture to CD61 expression using gates according to sort experiments. n = 2, Error bars, SD.; *p < 0.05, **p < 0.01, ***p < 0.001. (**d**) Cell count of AT- and SN-cells of CD34+CD61+ and CD34+CD61- sorted fresh cells after 5 days in ECM culture. n = 2, * = significance compared whole CD34+CD61+ and CD34+CD61- pre-sorted population including AT- and SN-cells, two-tailed t-test; Error bars, SD.; *p < 0.05, **p < 0.01, ***p < 0.001.

Our findings prompted us to investigate whether ITGβ3 expression is induced by ECM preparations or if the scaffold accumulates ITGβ3 expressing cells. Fluorescence-activated cell sorting (FACS) was used to sort either CD34^+^ITGβ3^−^ or CD34^+^ITGβ3^+^ cells from mobilized PB (Fig. 6a). After 5 days on ECM scaffolds, SN- as well as AT-cells were analyzed for CD34 and ITGβ3 expression. As expected, the sorted CD34^+^ITGβ3^+^ cells still exhibited strong ITGβ3 surface expression (55.4% ± 1.8%). However, only 31.2% ± 1.7% of SN CD34^+^ cells were positive for ITGβ3. Previously CD34^+^ITGβ3^−^ sorted AT- and SN-cells had upregulated ITGβ3 to levels of 29.9% ± 11.9% and 19.8% ± 7.35%, respectively (Fig. 6b,c). These data suggest that ITGβ3 surface expression is induced during ECM *ex-vivo* culture on a subset of HSPCs. Along this line, CD34^+^ITGβ3^+^ sorted cells showed an increased adhesion capacity, as compared to CD34^+^ITGβ3^−^ sorted cells. Up to 90% of CD34^+^ITGβ3^+^ cells were found to adhere to ECM proteins after 12 h (data not shown). However, sorted CD34^+^ITGβ3^+^ showed reduced proliferation and expansion of 1.2 ± 0.1 fold when cultured 5 days on ECM-scaffolds (Fig. 6d). CD34^+^ITGβ3^−^ cells proliferated significantly more, with a 2.9 ± 0.04 fold expansion (p < 0.0001), which is comparable to previously presented results with CD34^+^ cells isolated from PB (Fig. 1b). Thus, our data suggest that ITG β3 expression is induced by ECM preparations and is essential for effective adhesion and functional adaptation like migration and proliferation.

## Discussion

In this study, we have used decellularized ECM scaffolds derived from SCP-1 cells to mimic the hematopoietic stem cell niche *in vitro*. This mesenchymal cell line was generated from human MSCs overexpressing human telomerase reverse transcriptase to facilitate immortalization. These cells are known to display key features of MSCs like CD73, CD90 and CD105 surface marker expression and osteogenic, adipogenic and chondrogenic differentiation^19^. Additionally, constant ECM production and HSPC proliferation support was found. Comparable ECMs derived from human MSCs covalently bound to glass slices via poly-octadecene-alt-maleic anhydride and human fibronectin coating were demonstrated to have an average height of 1 µm. Compliance of these scaffolds was tested to be approximately 100 Pa^20^, which represents BM stroma properties^34^. Only 20% of PB-HSPCs were capable to primarily adhere to the standardized ECM preparations, which might reflect an induced loss of adhesion molecules due to G-CSF mobilization^40, 41^. However, stem cell expansion in the SN- and AT-cell fraction was enhanced by ECM scaffolds, probably due to the presentation and release of growth factors. Interestingly, AT-cells proliferated more, indicating adhesion related signaling as mediator of cell expansion. In addition, AT-cells showed morphological adaptations, depicted by elongated cell shape and migratory phenotype. Reichert *et al*. reported similar findings after 7 days of HSPC co-culture with either human or murine MSCs^39^. These observations point to the ECM as preliminary site of adhesion and migration of primary CD34^+^ HSPC.

BM homing and retention necessitates morphological adaptation towards migratory phenotype and are mainly promoted by MSCs releasing the chemokine SDF-1^42^. Circulating HSPCs recognize SDF-1 via CXC-Receptors (CXCR). These receptors are known to activate integrins such as LFA-1 (CD11a), VLA-4 (CD49d) and VLA-5 (heterodimer of CD49d and CD29), which leads to endothelial adhesion and trans-endothelial migration^43^. We found the most common one, CXCR4, to be expressed on SN- and AT-cells. However, in AT-cells CXCR4 seem to spread the whole cell cytoplasm, suggesting active SDF-1 turn-over with internalization of receptor/ligand complex^42^. We tested this internalization by immunostaining and demonstrated that SDF-1 was incorporated exclusively in AT-cells. Additionally, in AT-cells CXCR4 was polarized towards the ECM-scaffold. Similarly, in co-cultures with SCP-1 cells, here CXCR4 was shown to be polarized towards the confluent cell layer. These findings have been previously demonstrated for primary MSCs^44^ and probably indicate, that SDF-1 was released by SCP-1 cells, the chemokine was bound to ECM proteins and remains bio-active for up to 5 days culture period. In contrast, after addition of recombinant SDF-1 to cell culture medium, AT-cells lost their CXCR4 polarity and SN-cells showed internalization of the receptor/ligand complex. This indicates the active recognition of SDF-1, which nevertheless, does not lead to adhesion of SN-cells. Adhesion capacity to human MSC-layer was previously shown to be not altered by SDF-1/CXCR4 axis when CXCR4 was blocked using AMD3100^18^. However, less migration was found in trans-well assays showing that SDF-1 is recognized by HSPCs and facilitates retention and migration, but does not actively regulate adhesion promoting structures^28^. This is in accordance with our findings. Nevertheless, we focused on the incorporation of bio-active SDF-1 to demonstrate that ECM scaffolds closely mimic the chemokine gradient existing in the physiological BM niche. However, Sun *et al*. and Lai *et al*. have described SDF-1 to induce ITGαVβ3 expression in prostate cancer and chondrosarcoma cells, respectively, dependent on CXCR4 signaling^45, 46^. In line with these observations, culture of HSPCs on ECM scaffolds induces the expression of ITGβ3 on subsets of AT-cells to amounts present in the BM (Fig. S4d) and leads to a subsequent enrichment of ITGαVβ3^+^ cells. Freshly isolated cells were found mainly ITGβ3-negative. This might indicate an activation of ITGαVβ3 via SDF-1 when cells adhere to stroma and come in contact with incorporated SDF-1 to subsequently retain HSPCs in the niche.

ITGβ3 exclusively heterodimerizes with integrin αV or αIIb to form RGD recognizing focal contacts^36^. Both integrins were also upregulated in AT-cells. In HSPCs combinations of these ITGs are described as marker for long-term repopulating cells^14^ and as mediator of HSPC maintenance^3, 37^, and were correlated to properties of quiescent cells^13^. Additionally, we have detected a reduced migratory capacity of AT-cells, which is a key feature of stem cells when homing to or being retained in the BM^47^. Arguably, ITGβ3 expression could be a marker for commitment, since it is predominantly expressed in megakaryopoiesis^48, 49^ and erythropoiesis^50^. Additionally, ITGβ3 has been found to be essential for acute myeloid leukemia progression in mouse^51^.

Integrins were shown in the last decades to sense physical parameters and transduce mechanical cues into the cells to regulate fundamental functions such as migration, proliferation and differentiation^52^. In the context of HSPCs, maintenance and stem cell fate decisions, were found to be controlled via these contact-signaling machinery^53^. ECM culture induced focal contacts, necessitates filamentous actin association and therefore outside-in signaling^54^, which is also indicated by phosphorylated paxillin^55^. These structures transduce substrate physical cues into cells, via recruitment of other signaling molecules and directs lineage specification as nicely reviewed by Lee-Thedieck and Spatz 2014^56^. However, most of these studies were performed using single or combined protein coatings onto rigid substrates like glass or plastic or on hydrogels with different elastic moduli in the range of kilo Pascal hardly reaching BM physical properties of 0.24 to 25 kPa^57^. The elastic modulus of our ECMs ranged between 0.05 to 0.3 kPa closely reflecting BM properties^34^. Additionally, they consist of complex protein mixtures and therefore provide various interaction partners for HSPCs. Several ITGs were described as mechanosensors on HSPCs^58^, however, none of them was found to be differentially expressed in SN- or AT-cells. Interestingly, AT-cells adapt to ECM scaffold, as shown by their stiffer phenotype measured by RT-DC compared to SN-cells. This indicates that extracellular stiffness transduction into intracellular tension might be facilitated via combinations of ITGβ3, ITGαIIb and ITGαV. Bae *et al*. showed in mouse embryo fibroblasts recognition of stiff substrates (20 to 25 kPa) via focal adhesion kinase-cas-rac-signaling and tyr^118^ phosphorylation of paxillin. Moreover, the particular signaling cascade increased cell cycling, whereas cultivation on soft substrates (2 to 4 kPa) prevent cells from mitogen-stimulated cycling^59^. Additionally, Lee-Thedieck have demonstrated that the adhesion of CD34^+^ cells to fibronectin is enhanced by hard hydrogels (E > 38 kPa) compared to soft (*E* ≤ 20 kPa) scaffolds. Moreover, primary CD34^+^ showed increased random migration in the presents of SDF-1 when cultured on hard hydrogels^60^. We have found increased cycling, fast migration of up to 0.1 µm per second and membrane associated paxillin tyr^118^ phosphorylation. However, ECM scaffolds are more compliant, since ultra-soft scaffolds, like tropoelastin coatings on plastic dishes were shown as inducer of stem cell proliferation^23^. Therefore, our ECM approach represents a more complex set-up probably resembling the *in-vivo* situation. This hypothesis is supported by the detection of incorporated chemokines like SDF-1 and the provision of an extremely soft environment with the respective consequences on mechanotransduction. In contrast, thin ECM layers (1 µm in height) might not cover recognition of underlying glass slides and stiffness induced differentiation and migration. Therefore, a combination of hydrogels with different elastic moduli coated with decellularized ECMs could provide detailed insights into mechanosensing of HSPCs in a native BM ECM environment including storage of growth factors and other signaling molecules.

Various components of the niche environment regulate hematopoiesis, maintain HSPC in the marrow microenvironment and give rise to several niches increasing the complexity. By combining mechano-biological aspects and biochemical cues in one culture dish using BM-mimetics, we identified ITGβ3 induction to anchor cells to ECM proteins and give rise to migrating phenotype. Scaffold physical parameters might be transduced through outside-in signaling via ITGαVβ3 and lead to mechanical and functional adaptation, resulting in increased SDF-1 recognition and chemotactic migration. However, ECM tension transduction through focal adhesion contacts and the impact on stem cell commitment need to be further elucidated, enhancing the importance of stroma modeling *in vitro*.

## Material and Methods

### SCP-1 cell culture

SCP-1 cell line was generously provided by Matthias Schieker^19^. Cells were cultured in standard medium consisting of low-glucose DMEM (Life technology, USA) supplemented with 10% FCS (Biochrom, USA) at 37 °C and 5% CO2 in a humidified incubator. Cells were passaged once a week and medium was changed twice a week.

### Generation of surface immobilized SCP-1 derived ECM

Surface immobilization of ECMs and their subsequent characterization have been described in detail elsewhere^34^. Briefly, SCP-1 cells were seeded at a density of 1 × 10^4^ cells per cm² on poly-(octadecene-alt-maleic anhydride) (POMA) and human fibronectin (FN; 5 µg/cm²) coated glass slide. Medium was changed every second day. To yield cell-free ECM structures, cultures were decellularized at day 10 using warm double distilled water supplemented with 20 mM ammonium hydroxide (Sigma) and ECMs were obtained by gentle agitation for 10-min at room temperature. The resulting protein layers were three times washed with deionized water and twice with PBS containing calcium and magnesium. The obtained ECM scaffolds were used directly or stored in PBS containing calcium and magnesium for up to 4 weeks.

### CD34^+^ hematopoietic stem and progenitor cell purification and MSC co-culture

G-CSF (granulocyte colony-stimulating factor) mobilized peripheral blood was obtained from healthy donors after informed consent (ethical approval no. EK221102004, EK47022007). CD34^+^ HSPCs were purified from leukapheresis samples using CD34 antibody-conjugated magnetic beads according to the manufacturer’s instructions (Miltenyi Biotec). Purified CD34^+^ HSPCs were plated in 6 well plates containing ECM scaffolds and grown in CellGro medium (CellGenix) supplemented with 2.5 ng/ml stem cell factor (SCF), interleukin 3 (IL-3) and FMS-like tyrosine kinase 3 ligand (Flt-3) (all Miltenyi Biotec) for 5, 7, or 11 days without medium change. As negative control CD34^+^ HSPCs were plated in 6 well plates without ECM scaffold slides (plastic culture dish (PCD)-cells). For co-culture experiments SCP-1 cells were seeded at a density of 1 × 10^5^ cells per cm² and grown in DMEM supplemented with 10% FCS for 48 h, before adding 1 × 10^4^ cells per cm² freshly isolated CD34^+^ cells. SCP-1 / HSPC co-cultures were maintained in CellGro.

### Flow cytometry and fluorescent activated cell sorting

PCD cultured and SN-cells were harvested by three washes in PBS/5% FCS. AT-cells were detached by vigorous pipetting in PBS/5% FCS and harvested. Cells were stained for the following surface markers using fluorescently labeled antibodies according to manufactures protocol: CD34-alophycocyanin (APC; Miltenyi Biotec) CD41-fluorescein isothiocyanate (FITC; Immunotech), CD51-phycoerythrin (PE; BioLegend) and CD61-PE (BectonDickinson). Corresponding human immunoglobulin G controls were used. At least 10^4^ stained cells were measured by flow cytometry using FACScalibur or LSRII (both BectonDickinson) and were analyzed byFlowJo software version 7.6.5 (Tree Star). Fluorescent activated cell sorting was performed using FACSAria (BectonDickinson) (Fig. 6a). For dead cell exclusion 4′,6-Diamidin-2-phenylindol (DAPI) was used.

### Cell cycle analyses

BrdU assay was carried out following the manufacturer’s instructions for the BrdU Staining Kit for Flow Cytometry APC (affymetrix eBioscience) using 10 µM BrdU-staining solution. CellTraceTM CFSE Cell Proliferation Kit (Invitrogen) was used to detect HSPC generations according to manufactures instruction. Fresh isolated CD34^+^ cells were stained with Carboxyfluorescein succinimidyl ester and cultured either 5 d on Matrix or PCD. Cells were collected, washed and counterstained using CD34-APC AB. The number of cell divisions was quantified according to CFSE signal intensity using LSRII flow cytometer and FlowJo-software.

### Trans-well migration assay

A trans-well migration system (Neuroprobe) was used to test HSPC migration towards a gradient of SDF-1α (Stromal cell derived factor 1α (R&D)). This system provides a 96-well plate reversibly covered by a porous membrane (pore size = 5 μm). The cavities of the provided well plate were filled with 30 μl medium containing 100 ng/mL SDF-1α. Spontaneous migration rates were examined without adding SDF-1. After placing the membrane onto the plate, 60 μl of Cellgro medium containing equal amounts of cells (1.5 or 3 × 10^4^) was added onto the membrane corresponding to the cavities. Non-migrated cells were used as reverence. The total number of migrated cells was detected by MACS Quant cytometer (Miltenyi Biotec) and data were analyzed using FlowJo® software.

### Confocal laser scanning microscopy

PCD cultured or supernatant and adherent HSPCs on ECM preparations were fixed either with 4% paraformaldehyde for F-actin, CXCR4 and SDF-1 staining or methanol/aceton for integrin, vinculin and p-paxillin staining. Cells were permeabilized using PBS containing 0.1% Triton X-100 (T-PBS), blocked with T-PBS containing 10% FCS and 1% HSA (IF-Buffer) and incubated over night at 4 °C with Alexa Fluor 488 phalloidin for actin staining or antibodies as follows: monoclonal mouse-anti-human-ITGαVβ3 (Abcam) 1:200, polyclonal rabbit-anti-human-CXCR4 (Abcam) 1:500, polyclonal rabbit-anti-human-CXCL12/SDF-1 (Acris) 1:500, monoclonal mouse-anti-human-vinculin 1:500 and polyclonal rabbit-anti-human-p-paxillin (Tyr118) 1:50 (CellSignaling). After washing 3 times for 10 min with T-PBS secondary antibodies were incubated for 1 h room temperature as follows: polyclonal sheep-anti-rabbit-Cy3 (Sigma-Aldrich) 1:200 or polyclonal goat-anti-mouse-Cy2 (Dianova). After washing 3 times for 10 min with T-PBS DNA was counterstained with DAPI (Sigma-Aldrich). Cell evaluation was performed by confocal microscopy (Leica SP5).

### Real-time deformability cytometry

RT-DC was performed as described previously^29, 61, 62^. Briefly, fresh isolated CD34^+^ cells, Supernatant or Attached cells were harvested, washed with PBS and suspended in PBS containing 0.63% methylcellulose at a concentration of 1–2 × 10^6^ cells/mL. Suspension was drawn in a syringe and connected to a microfluidic chip made from Polydimethylsiloxane (PDMS) on a glass slide. The chip consisted of a central channel separated by two reservoirs. A second syringe, filled with MC-PBS, was used to hydrodynamically focus the cells inside the constriction of the chip, which was mounted to an inverted microscope. A syringe pump flushes cells through the channel at a constant flow rate of 0.06 µL/sec. A high-speed camera images the cells at the end of the constriction. Through the flow profile cells were deformed into a characteristic bullet like shape. In real-time cell cross-sectional area (Size [µm²]) and deformation were calculated and shown in scatter plot. The color code originates from a kernel density estimation and indicates the number of events (high density – low density). It has to be emphasized that deformation and size are not independent for RT-DC. This implies that for two cells of identical mechanical properties, the larger cell will always deform more. An analytical model combining Stokes fluid dynamics with linear elasticity allows to disentangle the relationship of size and deformation and to deduce material properties^61^. Statistical comparison of deformation was carried out with 1-dimensional linear mixed model analysis. One fixed and one random effect was considered, in order to analyze the difference between subsets of cells and to consider the replicates variance, respectively. P-values were determined by a likelihood ratio test, comparing the full model with a model lacking the fixed effect term.

### Live cell migration and integrin-αVβ3 blocking

To analyze the cell migratory behavior on ECM preparations SN-cells were removed by washing 3 times with PBS. Adherent HSPCs were live-cell imaged for 40 min using an inverted microscope Axiovert S100 (Carl-Zeiss). Every 30 sec an image was taken and cell migration was analyzed using Fiji-software and Chemotaxis and Migration Tool (ibidi). For functional integrin blocking attached cells were pre-incubated for 1 h before migration analysis either with 2 µg monoclonal mouse-anti-human-ITGαVβ3 (Abcam) antibody or appropriate IgG_1_ control in serum-free CellGro medium in 6 well plates.

### Statistics

Beside RT-DC measurements all data were analyzed using GraphPad Prism software (version 5.00 March 12, 2007 (GraphPad)) Mean values ± standard error (s.e.m.) or standard deviation (SD) are presented. Statistical significant differences using tests as described in figure legends are presented as *p ≤ 0.05; **p ≤ 0.01; ***p ≤ 0.001.

## Electronic supplementary material

Supplementary

Supplementary Movie 1
