# Supplementary material for: Bone marrow niche-mimetics modulate HSPC function via integrin signaling
Source: Sci Rep. 2017 May 31;7:2549. doi: 10.1038/s41598-017-02352-5 (PMC5451425; doi:10.1038/s41598-017-02352-5)
Supplement: Supplementary file 1 — Supplementary [file 41598_2017_2352_MOESM1_ESM.pdf]

Bone marrow niche-mimetics modulate HSPC function via integrin signaling

Martin Kräter[1,*], Angela Jacobi[2,*], Oliver Otto[3], Stefanie Tietze[1,2], Katrin Müller[1], David M. Poitz[4], Sandra Palm[1], Valentina M. Zinna[1], Ulrike Biehain[1], Manja Wobus[1], Triantafyllos Chavakis[5,6,7], Carsten Werner[8], Jochen Guck[2] and Martin Bornhauser[1,7]

[1] Medical Clinic I, University Hospital Carl Gustav Carus, Dresden, Saxony, 01307, Germany.
[2] Biotechnology Center, Technische Universität Dresden, Dresden, Saxony, 01307, Germany.
[3] Centre for Innovation Competence - Humoral Immune Reactions in Cardiovascular Diseases, University of Greifswald, Greifswald, Mecklenburg-Western Pomerania, 17489, Germany.
[4] Department of Internal Medicine and Cardiology, Technische Universität Dresden, Dresden, Saxony, 01307, Germany.
[5] Department of Clinical Pathobiochemistry, Technische Universität Dresden, Dresden, Saxony, 01307, Germany.
[6] Institute for Clinical Chemistry and Laboratory Medicine, Technische Universität Dresden, Dresden, Saxony, 01307, Germany.
[7] Center for Regenerative Therapies Dresden, Technische Universität Dresden, Dresden, Saxony, 01307, Germany.
[8] Leibniz Institute of Polymer Research Dresden, Max Bergmann Center of Biomaterials, Dresden, Saxony, 01307, Germany.

* These authors contributed equally.

Corresponding author

Martin Bornhauser
Medical Clinic I
University Hospital Carl Gustav Carus
Fetscherstraße 74
01307 Dresden
Germany
0049 351 458 4704
Martin.Bornhaeuser@uniklinikum-dresden.de

RunningTitle

BM-mimetics remodel HSPC function via ITGβ3

Supplementary information

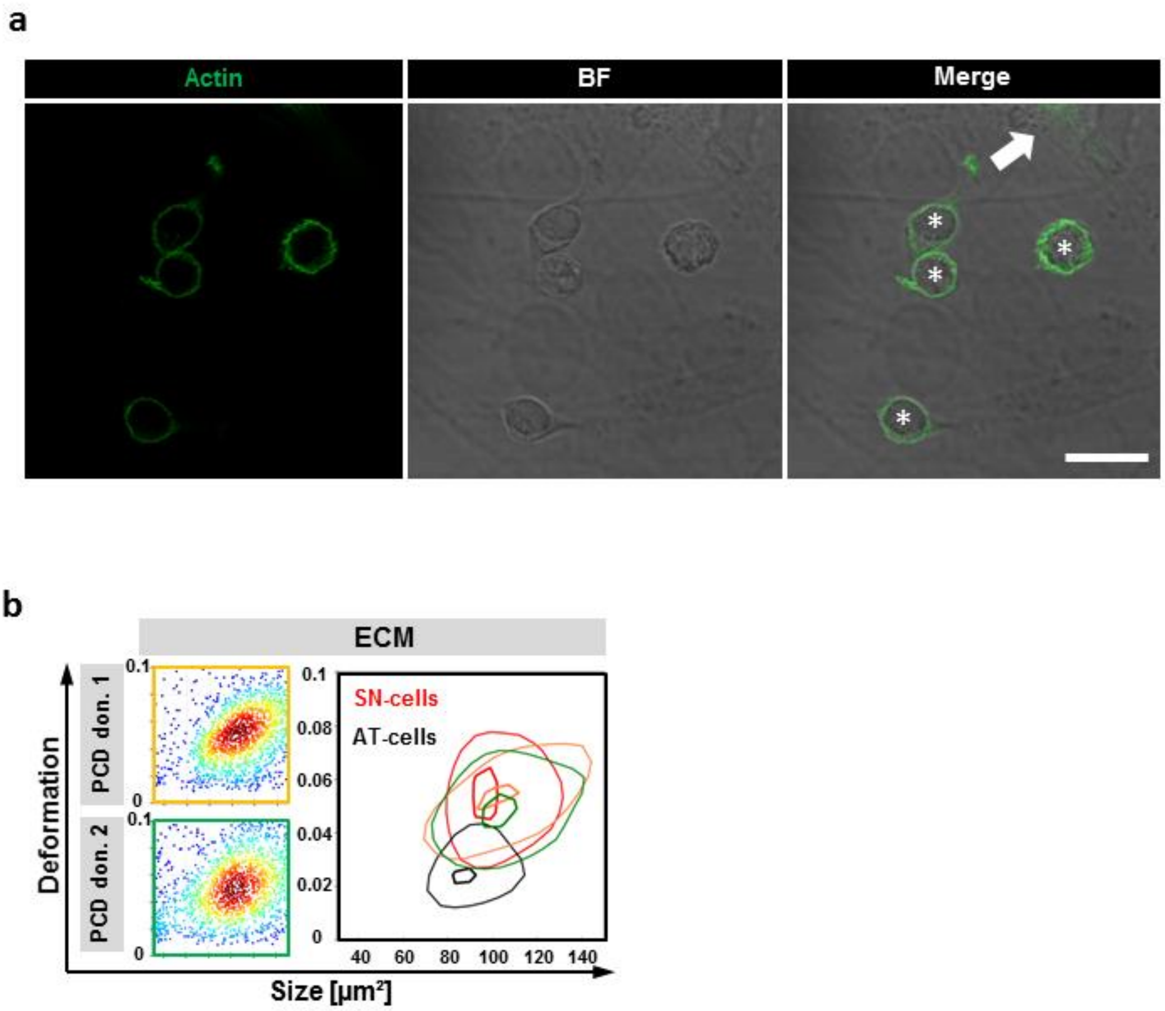

Supplement Figure 1. HSPC adhesion to SCP-1 cells in co-culture and mechanical fingerprint of PCD cultured HSPCs. (a) Freshly isolated HSPCs (104 cells per cm^2) were seeded to a confluent layer of SCP-1 cells. After 5 days in culture cells were fixed using 4 % PFA for 20 min. Phalloidin staining was applied and confocal microscopy was used to image filamentous actin. Confocal microscopy images show actin cytoskeleton of HSPCs (asterisks) adhered to SCP-1 cells. Arrow indicates actin staining of a SCP-1 cell. Bar = 10 µm. (b) HSPCs were cultured for 5 days in suspension culture using standard PCDs and measured to deformation and size in RT-DC. Two representative measurements are shown (PCD donor 1 and PCD donor 2). Contour-plot overlay highlights 95%- (inner line) and 50%- (outer line) density and shows donor 1 (orange) and donor 2 (green) overlaying with SN-cells (red). This plot shows that the PCD cells from donor 1, 2 and the SN cells are, more deformable than AT-cells (black).

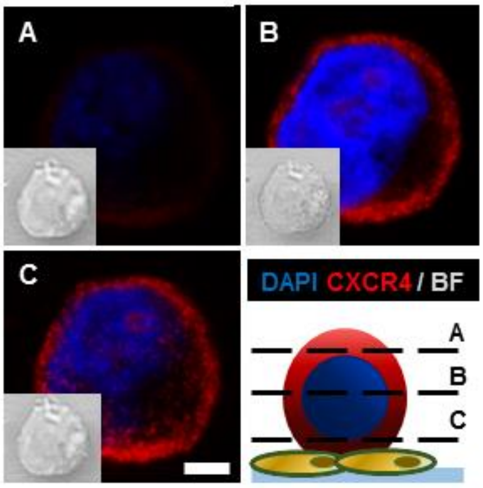

Supplement Figure 2. CXCR4 is polarized on HSPCs towards SCP-1 cell feeder layer. Fresh isolated HSPCs (104 cells per cm²) were seeded to a confluent layer of SCP-1 cells. After 5 days in culture cells were fixed using 4 % PFA for 20 min. Antibody staining was applied using α-CXCR4 antibodies. Confocal microscopy Z-stacks were used to determine expression pattern of CXCR4 on HSPCs. Representative images of one Z-stack show less CXCR4 signal on the upper part of the cell (away from SCP-1, A) and intense signal in the middle and lower part of the cell, B and C, respectively. Inserts show brightfield images. Schema shows technique. Bar = 5 µm.

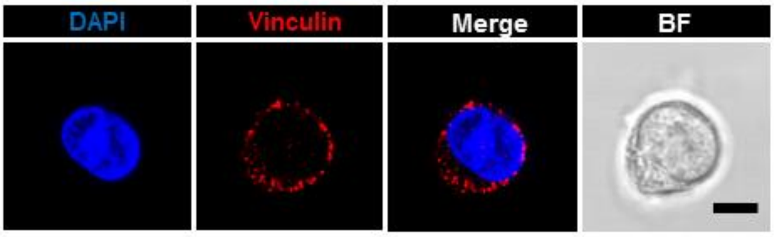

Supplement Figure 3. Vinculin expression on SN-HSPCs. (a) Fresh isolated HSPCs (104 cells per cm²) were seeded to an ECM scaffold. After 5 days in culture SN-cells were fixed using 4 % PFA for 20 min. Antibody staining was applied using a α-vinculin antibody. Confocal microscopy image show a representative cell positive for vinculin. Bar = 5 µm.

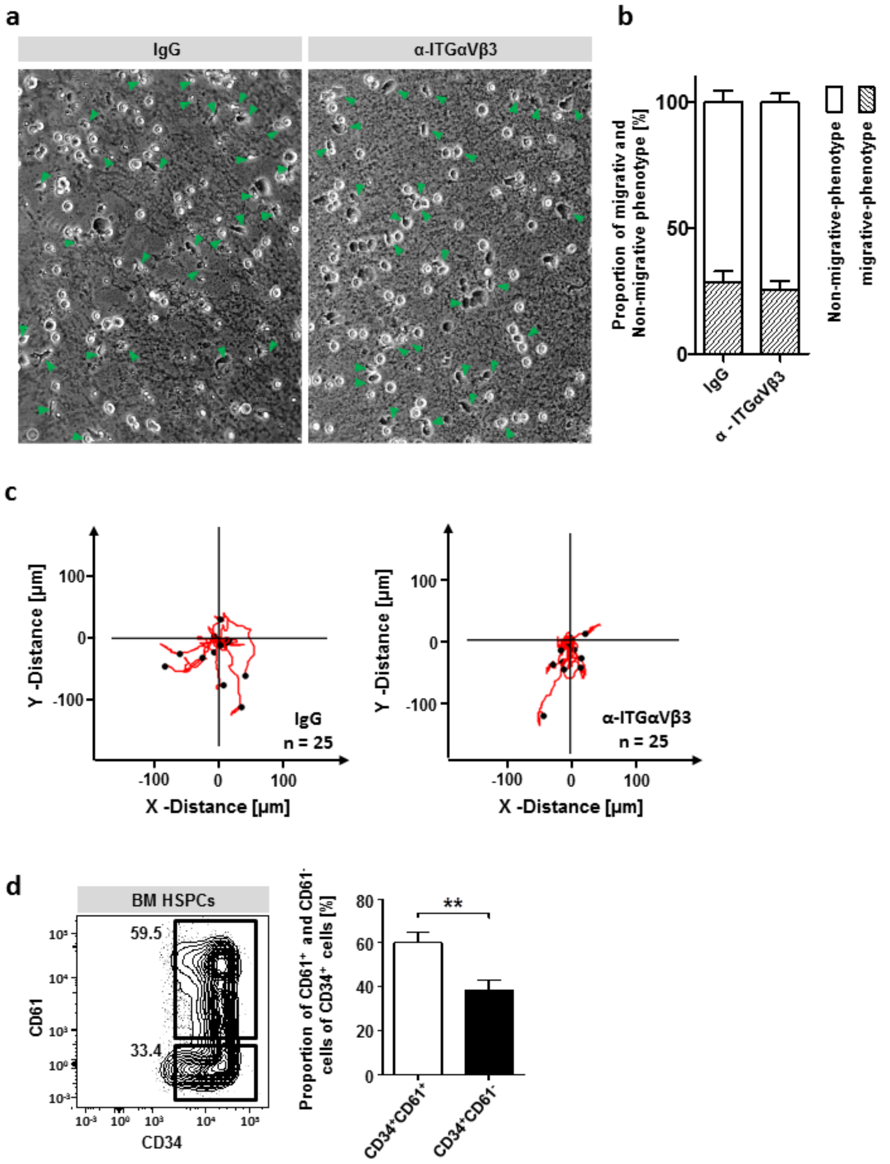

Supplement Figure 4. Blocking ITGαVβ3 do not lead to altered migratory phenotype but migration is reduced and BM-HSPC CD61 expression. (a) Fresh isolated HSPCs (104 cells per cm^2) were seeded to an ECM scaffold. After 5 days in culture cells were incubated one hour with a blocking antibody directed against ITGαVβ3 or a corresponding control IgG and imaged using brightfield microscopy. Elongated migratory cell shapes (arrowhead) or spherical non-migratory cell shapes (unmarked) were detected and counted. (b) Proportion of migratory and non-migratory phenotypes of 5 representative images of 5 donors either incubated one hour with a blocking antibody α-ITGαVβ3 or corresponding control IgG were calculated. No significant differences could be detected. (c) Cells were cultured 24 hours on ECMs and incubated one hour with α-ITGαVβ3 or IgG. Trajectory plots depict migration of 25 HSPCs for 45 minutes. (d) BM aspirates of healthy donors were AB stained and proportion of CD61+ cells out of CD34+ HSPCs was determined. n = 3, two-tailed t-test; Error bars, SD.; *$p < 0.05$, **$p < 0.01$, ***$p < 0.001$.
